# Public communication about public health where we really need to go

**DOI:** 10.1038/s41746-022-00574-0

**Published:** 2022-03-08

**Authors:** Megan L. Ranney, Stefanie Friedhoff

**Affiliations:** 1 School of Public Health, Brown University, Providence, RI USA; 2 Brown-Lifespan Center for Digital Health, Alpert Medical School, Brown University, Providence, RI USA

**Keywords:** Public health, Policy

Communication is a key part of the practice of public health and is a core competency required to be taught by schools of public health across the United States^1^. Part of this competency is the precept that communications about public health crises should be designed and delivered in a way that maintains trust and social cohesion, shares evolving science, and builds a broad base of support for measures that can save lives and livelihoods^2^.

During the COVID-19 pandemic, however, our world has faced unprecedented communications challenges, ranging from new communication modalities (like social media and other online media) to dramatically increased speed of knowledge creation and dissemination, to intentional campaigns of mis- and dis-information. A complex set of factors including politicization, distrust, division, and obfuscation—as well as basic errors in public health messaging—have occurred^3^. The public’s willingness to follow basic tenets of infection prevention and mitigation is at risk.

To do better in the future, we must rigorously evaluate the quality, quantity, and efficacy of public health communication during the COVID-19 pandemic. “The Language of Crisis: Spatiotemporal Effects of COVID-19 pandemic dynamics on health crisis communications by political leaders,” by Benjamin J. Mandl and Ben Y. Reis provides an important, but very preliminary, step at looking at the connections between public health communications by political leaders and public health outcomes^4^.

The Mandl and Ries paper used transcripts of governors’ speeches over a limited time frame (March–June 2020) to examine correlations between speech characteristics and COVID-19 rates. They applied basic natural language processing techniques to identify semantic categories and sentiments, and then correlated these language characteristics with COVID-19 case counts, both across space (across states) and across time (within states).

Their work yields some interesting insights.

First, during the earliest wave of the pandemic, governors’ speech patterns became simpler and more negative as COVID-19 cases increased. Higher COVID volumes also correlated with higher likelihood of governors’ using language about hospitals, prohibitions or policies, and religion. This finding is intellectually interesting, but not particularly informative for politicians, researchers, or communicators.

Second, high COVID volumes did not correlate with sentiments of helpfulness or community, nor with word patterns that discuss preventive measures. Indeed, governors’ language became more impersonal and less empowering as the crisis worsened: they increasingly spoke about “what is government doing for you” rather than “what can you do for yourself” or “what can you do for the common good.” We note that this tendency is the opposite of how healthcare professionals communicated during the COVID-19 crisis^5^, and is the opposite of what public health teaches us about behavior change. Given the crucial role the public plays in the collective response to a pandemic, an overfocus on government actions can result in a “we have no control” mindset and hinder resilience and community solidarity. The public’s responses to public health messaging in a crisis are heavily driven by pragmatism and self-efficacy^6^. Messaging that emphasizes community and individual action increases adherence with crisis response measures; reminders that one is part of a group reduces stress and anxiety; and people want to know what lies within their capacity to act and fits within the circumstances of their lives. The paper’s finding therefore points out a major area for improvement in public communication, and an opportunity to work with politicians.

Third, as is often true for the earliest work in a field, the paper creates more questions than it answers.

A critical limitation of this paper is that the authors evaluated speeches from only a very short period: the earliest months of the COVID-19 pandemic, when the world was virtually shut down and when we knew very little about the SARS-CoV2 virus. Our body of understanding and our set of tools to handle the pandemic is infinitely more complex today than it was during the period of this study. Our COVID-19 response is now also infinitely more politicized. We cannot help but wonder whether the observed findings would hold up across the initial wave of cases in more conservative states, from July–August 2020. We also wonder whether these findings would hold up across time, as all states changed their policies and measures. Ideally, this analysis would be repeated during summer 2020, winter 2020–2021, and again with the latest delta and new Omicron wave. We suspect that the correlations would be attenuated or even reversed during later time periods.

Another issue is that the findings seemed to be driven largely by a few states with both high volume of cases and high volume of gubernatorial speeches (e.g., New Jersey, New York, California, Rhode Island). It is difficult to know whether the correlations reflect politics, unique gubernatorial personalities, or actual case counts. Adjusting the analyses for political exigencies seems not just wise, but important.

Additionally, as the authors acknowledge, more advanced forms of linguistic analysis—to allow consideration of not just basic semantic categories such as “good” versus “bad”, but also more complex latent constructs—would be preferred. Others have already shown that this work is possible and we look forward to seeing it extended^7,8^.

Most importantly, this paper leaves us with the question of “so what?” The authors made the basic methodological choice to look backward, hypothesizing that caseloads influenced the governors’ words. Far more interesting, and useful, would be looking forward in time: examining the correlation between governors’ speech characteristics and future public health policy or (better yet) actual behavior and case counts.

Lastly, to get a more complete picture of how public speech and case counts intersect, we would also need to examine not just governors’ public speeches, but other forms of crisis communication, especially public health-led communications—and, ideally, public-driven online posts (Twitter, Facebook, and so on) as well. These forms of communication are perhaps more influential on—and more responsive to—local case counts than that of political leaders.

Many have opined about the failures of public health communications during the pandemic. This analysis provides one, albeit limited, insight into why and how we have erred. We hope that future analyses will both demonstrate the effect of our words and provide insights as to how we—as leaders, as communicators, and as a society—can do better.

## Reporting summary

Further information on research design is available in the Nature Research Reporting Summary linked to this article.

## Supplementary information

Reporting Summary

## References

[1] Council on Education for Public Health. Accreditation criteria schools of public health & public health programs. https://media.ceph.org/documents/2021.Criteria.pdf (2021).

[2] Bernhardt JM (2004). Communication at the core of effective public health. Am. J. Public Health.

[3] Sandman, P. M. Commentary: 8 things us pandemic communicators still get wrong. CIDRAP. https://www.cidrap.umn.edu/news-perspective/2021/12/commentary-8-things-us-pandemic-communicators-still-get-wrong (2021).

[4] Mandl, B. J. & Reis, B. Y. The language of crisis: spatiotemporal effects of COVID-19 pandemic dynamics on health crisis communications by political leaders. *npj Digit. Med*. **5**10.1038/s41746-021-00554-w (2022). 10.1038/s41746-021-00554-w PMC8748550 35013539

[5] Ojo, A., Guntuku, S. C., Zheng, M., Beidas, R. S. & Ranney, M. L. How health care workers wield influence through Twitter hashtags: Retrospective cross-sectional study of the gun violence and covid-19 public health crises. *JMIR Public Health Surveill.* **7**10.2196/24562 (2021). 10.2196/24562 PMC7790125 33315578

[6] Hyland-Wood B, Gardner J, Leask J, Ecker UKH (2021). Towards effective government communication strategies in the era of COVID-19. Humanit Soc. Sci. Commun..

[7] Guntuku, S. C., Purtle, J., Meisel, Z. F., Merchant, R. M. & Agarwal A. Partisan Differences in Twitter language among United States legislators during COVID-19: cross-sectional study. *J. Med. Internet Res.*10.2196/27300 (2021). 10.2196/27300 PMC8176946 33939620

[8] Lyu, J. C., Han, E. L. & Luli, G. K. Covid-19 vaccine–related discussion on Twitter: topic modeling and sentiment analysis. *J. Med. Internet Res*. **23**10.2196/24435 (2021). 10.2196/24435 PMC8244724 34115608

